# Evolution of metabolic alterations 5 Years after early puberty in a cohort of girls predisposed to polycystic ovary syndrome

**DOI:** 10.1186/s12958-017-0275-0

**Published:** 2017-07-24

**Authors:** Soren Harnois-Leblanc, Andréanne Trottier, Samuel Leblanc, Marie-Claude Battista, David H. Geller, Jean-Patrice Baillargeon

**Affiliations:** 1 Division of Endocrinology, Department of Medicine, Faculty of Medicine and Health Sciences, Université de Sherbrooke, 3001, 12e Avenue Nord, Sherbrooke, J1H 5N4 Québec Canada; 2 Research Center, Centre Hospitalier Universitaire de Sherbrooke, 3001, 12e Avenue Nord, Sherbrooke, J1H 5N4 Québec Canada; 3 Department of Pediatrics, Cedars-Sinai Medical Center, 8700 Beverly Boulevard, Los Angeles, 90048-1865 California USA

**Keywords:** Polycystic ovary syndrome, Daughters, Puberty, Insulin sensitivity, Non-esterified fatty acids, Glucose homeostasis

## Abstract

**Background:**

We and others have observed that young girls predisposed to polycystic ovary syndrome (PCOS) display defective insulin sensitivity, *beta*-cell function and non-esterified fatty acids (NEFA) suppressibility during early pubertal years, compared to controls. Our objective is to assess whether these differences in glucose and NEFA metabolisms persist after 5 years in late/post-puberty.

**Methods:**

We conducted a prospective cohort study between 2007 and 2015 with 4–6 years of follow-up in an academic institution research center. We compared 8 daughters and sisters of PCOS women (PCOSr) to 8 age-matched girls unrelated to PCOS (±1.5 years). Girls were assessed initially at 8–14 years old and re-assessed after a median follow-up of 5.4 years, at 13–21 years old. Our main measures were a frequently sampled intravenous glucose tolerance test (FSivGTT)-derived insulin sensitivity (IS) and *beta*-cell function (disposition index, DI_FSivGTT_); and indices of NEFA suppression during FSivGTT (log_n_-linear slope of NEFA and T_50_ of NEFA suppression).

**Results:**

At follow-up, both PCOSr and controls had similar results: IS = 3.2 vs 3.4 (*p* = 0.88), DI_FSivGTT_ = 1926 vs 1380 (*p* = 0.44), log_n_-linear slope = −0.032 vs −0.032 (*p* = 0.88) and T_50_NEFA = 18.1 vs 20.8 min (*p* = 0.57). IS, DI_FSivGTT_ and NEFA suppressibility were stable in PCOSr after 5 years, but decreased significantly in controls (all *p* < 0.05).

**Conclusions:**

Impaired metabolism observed during early puberty in girls predisposed to PCOS remains stable after 5 years whereas control girls deteriorated their metabolic parameters. Therefore, both groups become comparable in late/post-puberty. Early puberty may thus represent a window during which metabolic alterations are transiently apparent in girls at risk of PCOS.

## Background

Polycystic ovary syndrome (PCOS) is a common disorder, affecting 6–12% of women of reproductive age [1, 2]. It is defined as clinical and/or biochemical hyperandrogenism with ovarian dysfunction such as oligo-anovulation and/or polycystic ovaries, excluding any other androgen excess disorder [3]. Women suffering from PCOS will present increased risk of cardiometabolic morbidities, with insulin resistance detected in 50 to 80% of the cases [4]. A greater prevalence of impaired glucose tolerance and type 2 diabetes is observed compared to women without PCOS for the same age and weight [5].

PCOS and its cardiometabolic features are more common in families. Twenty to 40% of daughters and sisters of women with PCOS will develop the syndrome [6]. Several studies have shown that first degree relatives of women with PCOS display higher visceral adiposity, insulin resistance and associated hyperinsulinemia [7–10], both in women and men. Daughters or sisters of women diagnosed with PCOS develop abnormalities in glucose metabolism during early pubertal years, before developing clinical features of the syndrome [11–15]. In fact, between 8 and 12 years of age, they display lower insulin sensitivity and *beta*-cell dysfunction when compared to age- and body mass index (BMI) -matched girls unrelated to PCOS [14, 15]. Our group also observed lower non-esterified fatty acid (NEFA) suppression in these girls predisposed to PCOS [15]. However, it is still unknown how these metabolic defects evolved during and after puberty in comparison to the normal physiological changes associated with puberty. It has indeed been shown that insulin sensitivity decreases significantly during puberty in girls [16].

Early appearance of metabolic disruptions before development of PCOS per se in predisposed girls suggests that emergence of PCOS could be influenced by metabolic factors that enhance ovarian hyperandrogenism, perhaps as a primary defect or perpetuating factor. Hyperinsulinemia is known to alter steroidogenesis in ovarian theca cell resulting in decreased levels of progesterone and increased testosterone [17]. Insulin will also reduce hepatic synthesis of sex-hormone binding globulin (SHBG) and subsequently raise circulating free testosterone levels [17]. Furthermore, altered NEFA suppression implies the resistance of adipocytes to insulin, which can enhance NEFA spillover to visceral organs. Muscle, liver and *beta*-cell dysfunction following overexposure to NEFA (lipotoxicity) has been observed in the development of insulin resistance and type 2 diabetes [18]. There is yet no evidence of a lipotoxic effect on androgen-secreting organs, but experimental overexposure to NEFAs has been shown to increase androgen levels in vivo [19] and in vitro [20]. These studies suggest that lipotoxicity could induce androgen overproduction in androgen-secreting glands.

Identification of early defects that precede PCOS development in predisposed girls is vital to our understanding of the pathogenesis of the syndrome, and to more accurately identify girls who could benefit from intensified follow-up and/or preventive interventions, such as lifestyle management. Keeping in mind this overarching objective, our first step was to assess how metabolic abnormalities evolved until late- and post-puberty in daughters or sisters of women with PCOS as compared to girls unrelated to PCOS. Accordingly, the specific aim of this controlled cohort study was to evaluate whether the metabolic abnormalities previously identified [15] during early puberty in daughters and sisters of women with PCOS persist 5 years later, in late puberty or early after puberty.

## Methods

### Design

We conducted a controlled cohort study at the research center of the Centre hospitalier universitaire de Sherbrooke (CHUS), an academic center.

### Study participants

Participants were girls at risk of PCOS (PCOS relatives, PCOSr) and control girls previously evaluated between 8 and 12 years old by our group [15]. Probands with PCOS were diagnosed and followed by Dr. Jean-Patrice Baillargeon at the Reproductive Endocrinology Clinic of the CHUS*.* Girls from the control group were recruited at the outpatient pediatric endocrine clinic, where they were followed for a stable condition, and were matched to PCOSr on the basis of age (±1.5 years).

Assignment of PCOS diagnosis in probands was based on the The Androgen Excess and PCOS Society criteria [3]: oligomenorrhea (≤8 menstrual periods/year) or confirmed oligo-anovulation; clinical or biochemical signs of hyperandrogenism (acne, hirsutism, serum total testosterone >2.7 nmol/L or calculated free testosterone >25 pmol/L) [21]; and exclusion of secondary causes, i.e. non-classical congenital adrenal hyperplasia, abnormal thyroid function, hyperprolactinemia, evidence of androgen-secreting tumours, Cushing’s syndrome or acromegaly, or the use of medications known to affect levels of testosterone or 17OHPg within 3 months of testing.

PCOSr and controls girls of the original cohort were invited for follow-up 5 years after their initial research visit. Exclusion criteria from baseline were again verified: precocious puberty, medication known to affect glucose homeostasis such as insulin sensitizers, having diabetes or other uncontrolled metabolic disorder or following a highly-restrictive diet or intense physical activity program. Contrarily to the baseline study, we allowed participants to use oral contraceptives because it was unethical to suspend their contraception method for few months.

### Clinical assessment of subjects

Pubertal stages were determined according to Tanner criteria [22] and hirsutism according to the modified Ferriman-Gallway score [23]. The following anthropometric parameters were measured: weight, height, waist circumference, hip circumference, body fat mass and lean mass percentage using foot-to-foot bio-impedance (TANITA, Arlington Heights, IL, USA). BMI was calculated by dividing weight (kg) by the squared height (m^2^). BMI z-scores and percentiles were obtained from the Center for Disease Control and Prevention growth charts [24] (the maximum age of 19.99 years was used for participants ≥20 years old). Obesity was defined as an age and sex adjusted BMI z-score ≥ 95th percentile [25]. Waist circumference was measured between the inferior costal margin and the iliac crest in standing position. Hip circumference was taken at the level of the femoral trochanters. Waist circumference (WC) is strongly associated with cardiometabolic risks [26] and can be adjusted for stature in child with the waist-to-height ratio (WHtR). WC and WHtR z-scores were obtained using LMS tables developed by Sharma et al. [27]. These indices of adiposity are more strongly associated with adverse metabolic outcomes than BMI [27].

### Experimental protocol

Participants were assessed after a 12-h overnight fast for two research visits, same as the baseline study [15]. The 1st visit included anthropometric measures and physical examination described above, fasting blood sampling and a 2-h 75 g oral glucose tolerance test (OGTT). One month later, a 3½-hour insulin-modified, frequently sampled intravenous glucose tolerance test (FSivGTT) was performed (visit 2). In contrast to baseline visits, there was no measurement of sex hormones, considering the use of hormonal contraception in a large proportion of our study population at follow-up. Also, participants taking hormonal contraception were studied during the period without hormones in order to minimize their potential impact on metabolism.

#### Oral glucose tolerance test (OGTT)

Sampling for glucose, insulin and NEFA were collected at times −15, −5, 0, 15, 30, 60, 90 and 120 min after glucose load (40 g/m^2^ body surface area). Fasting values were the mean of times −15, −5 and 0 min. Areas under the curve (**AUC**) for glucose and insulin were calculated. Total adiponectin, leptin and triglycerides concentrations were assessed at time 0 and 120 min. The following indices were calculated from the OGTT: Mastuda insulin sensivity index (**ISI**_**Matsuda**_ = 10,000/[square root (fasting glucose (mg/dL) × fasting insulin (μU/mL)) × (AUC glucose (mg/dL) × AUC insulin (μU/mL))]) [28, 29], corrected insulin response to glucose at 30 min (**CIR30** = [insulin 30 min (μU/mL)]/[glucose 30 min (mg/dL)–70]) [30] and the corresponding **disposition index** (**DI**_**OGTT**_ = ISI_Matsuda_ × CIR30). The disposition index reflects insulin secretion adjusted for the level of insulin sensitivity and is therefore an estimation of *beta*-cell function [31].

After a glucose load, the insulin surge suppresses circulating NEFA levels. Indices of insulin-induced suppression of NEFA include the slope of the log-linear decrease of NEFA levels during the OGTT (**Ln(NEFA) Slope)** [32], and the T50 of NEFA suppression. **T50**_**NEFA**_ is defined as the time required for 50% suppression of NEFA levels at time 0. We calculated the AUC of insulin during the same period using the trapezoidal method, which reflects total tissue exposure to insulin. Since NEFA suppression is regulated by circulating insulin levels, Ln(NEFA) Slope was corrected for insulin with the ratio **Ln(NEFA) Slope/AUC**_**insulin**_.

#### Frequently sampled intravenous glucose tolerance test (FSivGTT)

Fasting samples were taken at time − 15, −5 and 0 min before the bolus of dextrose at time 0 (11.4 g/m^2^ of body surface area). An intravenous bolus of 0.02 U/kg insulin (Humulin Regular, Eli Lilly) was administered at time 20 min [28]. A total of 26 samples of glucose, insulin and NEFA were taken over 210 min. Dynamic indices of glucose metabolism from glucose and insulin data were obtained using the minimal model of Bergman (MINMOD computer program version Millennium 6.02, Richard N. Bergman, 2004) [33]: insulin sensitivity (**IS**_**FSIVGTT**_) [28], insulin secretion (acute insulin response to glucose **(AIRg)**) [34] and the corresponding disposition index (**DI**_**FSIVGTT**_ = IS_**FSIVGTT**_ × AIRg) [35].

NEFA level suppression was estimated with the natural logarithm slope of NEFA levels (**Ln(NEFA) Slope**) during the endogenous insulin-induced NEFA suppression phase of FSivGTT (time 0 to 20 min). We adjusted for area under the insulin curve calculated for the first 20 min of FSivGTT, using the ratio **Ln(NEFA) Slope/AUC**_**insulin**_. The time for 50% suppression of NEFA levels (**T50**_**NEFA**_) was assessed regardless of FSivGTT phases, but usually occurred during the first 20 min and thus reflected the effect of endogenous insulin [15].

### Assays

Plasma glucose concentrations were assayed by the glucose hexokinase technique (Beckman Coulter, Brea, CA, USA). Human insulin was measured by an electrochemiluminescence immunoassay (ECLIA; Roche Diagnostics, Indianapolis, IN, USA). Leptin levels were measured by ELISA (Luminex Technology; EMD Millipore, Billerica, MA, USA). Total adiponectin levels were measured by radioimmunoassay (EMD Millipore, Billerica, MA, USA). NEFA and triglyceride concentrations were assayed using enzymatic colorimetric assays (Wako Chemicals, Richmond, VA, USA).

### Statistical analyses

Results are expressed as medians with their interquartile range (25th and 75th). Groups were compared using Mann-Whitney tests. We used paired Wilcoxon tests to compare follow-up and baseline results. Since the change in WHtR z-score (from baseline to follow-up) was the adiposity measurement that was the most significantly different between groups, we adjusted group comparisons for changes in WHtR z-score using multiple linear regressions. Due to our small sample size, it was not possible to adjust for another factor. Statistical analyses were done with SPSS© 20.0 software (IBM©, Armonk, NY, USA). The level of significance was determined at 5%.

## Results

From the 9 girls having a first-degree relative diagnosed with PCOS and the 10 girls unrelated to PCOS recruited at baseline, we were able to reassess 7 girls from the PCOS relative group and 8 girls from the control group. Two PCOSr and one control girls withdrew between baseline and follow-up, and one control girl was lost to follow-up. A new PCOSr girl did a baseline visit just after the end of the baseline article [15] and had follow-up visit 5 years later. Hence, we report follow-up results for 8 PCOSr and 8 control girls who we were able to match for age (±1.5 years).

As shown in Table 1, the median time to follow-up was 5.4 years for the entire group. Most girls were in their late or post-pubertal years (Tanner stage ≥ 4) and post-menarchal. None of the girls presented hirsutism (Ferriman-Gallwey scores = 0). Hormonal contraceptive use was the same in both groups (37.5%). There were no differences in anthropometric measurements between groups at follow-up. Median BMI z-score was within normal range, but 2 girls in each group were obese (>95th percentile). Median WC and WHtR z-scores were between 15th and 35th percentiles, indicating normal degree of adiposity for age. One girl in the PCOSr group had WC and WHtR z-scores over the 95th percentile, but none in the control group.

**Table 1:** Clinical and anthropometric characteristics at 5-year follow-up in controls and PCOSr girls

	Controls (*n* = 8)	PCOSr (*n* = 8)
Age (years)	17.6 (14.6–20.2)	17.5 (14.4–20.1)
Duration of follow-up (years)	5.4 (5.3–6.0)	5.6 (4.6–6.6)
Tanner stage ≥4 (n, %)^a^	8 (100.0)	7 (87.5)
FG score > 8 (n, %)	0 (0.0)	0 (0.0)
Post-menarche (n, %)	7 (87.5)	7 (87.5)
Hormonal contraceptives (n, %)	3 (37.5)	3 (37.5)
Weight (kg)	57.4 (45.7–77.6)	58.5 (54.2–73.9)
BMI (kg/m^2^)	22.4 (19.1–27.8)	22.7 (21.5–30.8)
BMI Z-score	0.26 (−0.96–1.52)	0.63 (0.02–1.71)
Obese (n, %)	2 (25)	2 (25)
Fat percentage (%)	27.6 (13.4–37.9)	29.0 (24.1–35.4)
Waist circumference Z-score	−0.98 (−1.50–0.50)	−0.38 (−1.25 – −0.38)
WHtR Z-score	−0.88 (−1.06–0.54)	−0.41 (−1.04–0.57)

Continuous values are expressed as median (25th–75th percentile) and compared with Mann-Whitney test

Categorical values are expressed as n (%) and compared with Chi-Square or Fisher’s exact test

*BMI* Body mass index, *WHtR* Ratio of waist circumference (cm) to height (cm);

*FG score* Ferriman-Gallwey score

^a^Tanner stage according to the highest score of its three components: pubic hair, axillary hair and breast development

There were no significant differences between groups according to *p* values >0.05

**Fig. 1:**
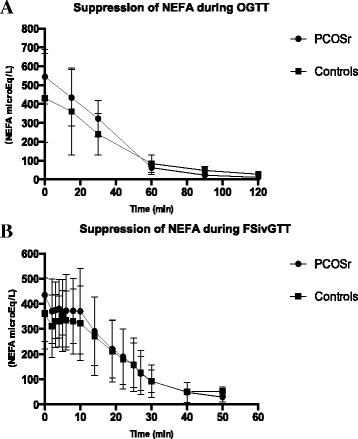
(Figure provided in a separate file). Insulin-induced suppression of non-esterified fatty acids (NEFA) during OGTT (**a**) and FSivGTT (**b**) in girls related to polycystic ovary syndrome (PCOSr, black circles) and control girls (Controls, black squares). Data are means ± SD

Table 2 shows fasting and 2 h results from the OGTT. Two-hour NEFA levels were significantly lower in PCOSr vs control girls (*p* = 0.003). Other markers were equivalent between PCOSr and control girls at follow-up. Fig. 1 presents similar curves of NEFAs levels across time for PCOSr and controls during OGTT and FSivGTT at follow-up.

**Table 2:** Metabolic measures at 5-year follow-up, during fasting and 2-h OGTT, after 75 g glucose overload

	Controls (*n* = 8)		PCOSr (*n* = 8)	
	Fasting	2 h	Fasting	2 h
Glucose (mmol/l)	5.0 (4.6–5.3)	5.7 (5.1–7.3)	4.6 (4.4–4.8)	5.8 (5.3–6.8)
Insulin (pmol/l)	70 (38–109)	290 (114–506)	65 (32–75)	361 (239–643)
NEFA (μEq/L)	395 (234–616)	25 (17–41)	582 (391–661)	11 (4–14)^a^
Triglycerides (mmol/l)	1.2 (0.7–1.6)	-	0.7 (0.6–0.9)	-
Total adiponectin (mg/l)	9.8 (6.9–12.2)	10.4 (6.9–13.6)	10.7 (8.5–15.4)	11.8 (8.7–15.7)
Leptin (*μ*g/l)	8.4 (2.9–14.3)	7.5 (2.5–17.6)	8.6 (4.4–15.4)	6.1 (3.6–21.6)

Values are expressed as median (25th–75th percentiles) and compared between groups with Mann-Whitney test

^a^Significant difference between groups, *p* value = 0.003

To convert values for glucose to mg/dL, multiply by 18; for insulin to μU/mL, multiply by 0.17; for triglycerides to mg/dL, multiply by 88.50

*NEFA* Non-esterified fatty acids

Table 3 presents metabolic parameters derived from glucose, insulin and NEFAs measured during the OGTT and FSivGTT. Indices of insulin sensitivity and *beta*-cell function were similar between PCOSr and controls at follow-up. NEFA suppressibility during OGTT was higher in PCOSr vs control girls, as shown by a steeper log_n_-linear slope of NEFA suppression, although this was no longer true after correction for insulin levels (AUC_Insulin_). Furthermore, the log_n_-linear slope of NEFA during FSivGTT and the T50_NEFA_ from OGTT and FSivGTT were similar between PCOSr and controls.

**Table 3:** Calculated metabolic parameters at 5-year follow-up, based on OGTT and FSivGTT measures

Conditions	Metabolic parameter	Calculated parameter	Controls (*n* = 8)	PCOSr (*n* = 8)	p
OGTT	Insulin sensitivity	ISI_Matsuda_	3.6 (2.4–7.3)	3.8 (2.6–6.0)	0.798
	Insulin secretion	CIR30	1.1 (0.6–1.3)	1.2 (0.8–1.7)	0.574
	β-cell function	DI_OGTT_	2.7 (2.3–4.7)	4.4 (4.1–6.1)	0.195
	NEFA suppressibility	Log_n_-linear slope	−0.028 (−0.030 – −0.018)	−0.040 (−0.044 – −0.036)	<0.001
		Log_n_-linear slope / AUC_insulin_	−0.58 (−1.02 – −0.23)	−0.81 (−1.29 – −0.51)	0.195
		T50_NEFA_ (min)	40.6 (28.3–48.5)	34.1 (24.4–47.0)	0.645
FSivGTT	Insulin sensitivity	IS_FSivGTT_	3.4 (1.8–3.6)	3.2 (2.2–5.3)	0.878
	Insulin secretion	AIRg	790 (343–813)	496 (361–997)	0.959
	β-cell function	DI_FSivGTT_	1380 (1122–2563)	1926 (1376–2264)	0.442
	NEFA suppressibility	Log_n_-linear slope	−0.032 (−0.040 – −0.024)	−0.032 (−0.088 – −0.018)	0.878
		Log_n_-linear slope / AUC_insulin_	−4.33 (−6.42 – −3.14)	−3.33 (−7.64 – −2.67)	0.505
		T50_NEFA_ (min)	20.8 (16.2–26.9)	18.1 (16.1–23.9)	0.574

Values are expressed as median (25th–75th percentiles) and compared with Mann-Whitney test

*AIRg* Acute insulin response to glucose, *AUC* Area under the curve, *CIR30* Corrected insulin response at 30 min, *FSivGTT* Frequent sampling intra-venous glucose tolerance test, *DI* Glucose disposition index, *DI*_*OGTT*_ ISI_Matsuda_ × CIR30, *DI*_*FSivGTT*_ IS_FSivGTT_ × AIRg, *IS*_*FSivGTT*_ Insulin sensitivity determined during the FSivGTT, *ISI* Insulin sensitivity index, *Log*_*n*_ *-linear slope* Slope of Ln of NEFA concentrations (between time 0 to 20 min for the FSivGTT), *Log*_*n*_ *-linear slope /AUC*_*Insulin*_ Ratio of the Slope of Ln of NEFA concentrations to the area under the insulin curve ×1000 (between time 0 to 20 min for the FSivGTT), *NEFA* Non-esterified fatty acid, *OGTT* Oral glucose tolerance test, and *T50*_*NEFA*_ Time to suppress 50% of NEFA baseline levels

Variations of selected anthropometric and metabolic parameters during follow-up are shown in Table 4. Adiposity measures did not vary significantly between baseline and follow-up, for either PCOSr or controls, except the WHtR that decreased in PCOSr and increased in control girls with a near significant between-group difference. Insulin sensitivity and *beta*-cell function derived from the OGTT and FSivGTT decreased significantly in control girls (OGTT: *p* = 0.012 and 0.017, FSivGTT: *p* = 0.012 and 0.012, respectively), but not in PCOSr girls. These changes were significantly different between groups. Moreover, changes in DI_OGTT_, IS_FSivGTT_ and DI_FSivGTT_ remained significantly different between groups after correction for changes in WHtR z-score (*p* = 0.024, 0.030 and 0.034, respectively). Both OGTT-derived NEFA suppressibility indices significantly decreased in control girls (log_n_-linear slope: *p* = 0.017 and T50_NEFA_: *p* = 0.028). The log_n_-linear NEFA slope significantly improved in PCOSr girls (*p* = 0.028), such that the change in log_n_-linear NEFA slope was significantly different between groups. FSivGTT-derived NEFA suppressibility decreased in controls according to a longer T50_NEFA_ (*p* = 0.025), but remained stable in PCOSr girls. These changes were not significantly different between groups.

**Table 4:** Variations of selected metabolic parameters between follow-up and baseline and compared between PCOSr and controls girls

*Conditions*	*Metabolic parameter*	*Calculated parameter*	Controls (*n* = 8)	PCOSr (*n* = 8)	*p*
Duration of follow-up (years)			5.4 (5.3–6.0)	5.6 (4.6–6.6)	1.000
Anthropometric measures	Adiposity	BMI Z-score	0.81 (−0.19–1.32)	−0.08 (−0.43–0.17)	0.105
		WHtR Z-score	0.50 (−0.18–1.07)	−0.43 (−0.93–0.21)	0.050
OGTT	Insulin sensitivity	ISI_Matsuda_	−7.7 (−12.2 – −2.2)^a^	−0.7 (−3.9–2.5)	0.050
	β-cell function	DI_OGTT_	−4.0 (−8.6 – −1.4)^a^	0.2 (−1.6–3.4)	0.010*
	NEFA suppressibility	Log_n_-linear slope	0.023 (0.016–0.027)^a^	−0.006 (−0.014 – −0.001)^a^	0.001
		T50_NEFA_ (min)	18.2 (3.4–33.5)^a^	10.5 (−0.6–17.4)	0.161
FSivGTT	Insulin sensitivity	IS_FSivGTT_	−7.3 (−11.6 – −4.6)^a^	−0.5 (−2.0–0.3)	0.001*
	Insulin secretion	AIRg	274 (89–506)^a^	190 (56–328)^a^	0.442
	β-cell function	DI_FSivGTT_	−689 (−1693 – −333)^a^	614 (−151–1233)	0.003*
	NEFA suppressibility	Log_n_-linear slope	0.015 (0.003–0.033)	−0.001 (−0.067–0.012)	0.083
		T50_NEFA_ (min)	9.6 (3.0–13.9)^a^	−1.3 (−6.0–6.5)	0.065

Values are expressed as median (25th–75th percentiles) and compared with Mann-Whitney test

^a^Significant change between baseline and follow-up: *p* < 0.05 analyzed with paired Wilcoxon test

*p < 0.05 after correction for the difference in WHtR Z-score between follow-up and baseline

*AIRg* Acute insulin response to glucose, *FSivGTT* Frequent sampling intra-venous glucose tolerance test, *DI* Glucose disposition index, *DI*_*OGTT*_ ISI_Matsuda_ × CIR30, *DI*_*FSivGTT*_ IS_FSivGTT_ × AIRg, *IS*_*FSivGTT*_ Insulin sensitivity determined during the FSivGTT, *ISI* Insulin sensitivity index, *Log*_*n*_ *-linear slope* Slope of Ln of NEFA concentrations (between time 0 to 20 min for the FSivGTT), *NEFA* Non-esterified fatty acid, *OGTT* Oral glucose tolerance test, and *T50*_*NEFA*_ Time to suppress 50% of NEFA baseline levels and *WHtR* Ratio of waist circumference (cm) to height (cm)

## Discussion

This study is the first report on the evolution of metabolic characteristics over 5 pubertal years in PCOS first-degree relatives and control girls matched for age. Interestingly, we found that alterations in glucose metabolism and NEFA suppressibility observed in girls at risk of developing PCOS during early/mid puberty [15] remained essentially stable over 5 years, into late and post-puberty. On the other hand, control girls’ insulin sensitivity, *beta*-cell function and NEFA suppressibility decreased significantly during the same period; such that theses parameters decreased significantly more in controls vs PCOSr girls, even after correction for changes in central adiposity (WHtR z-score). Accordingly, we observed that PCOSr and control girls display remarkably similar glucose homeostasis and NEFA suppressibility in late puberty or soon after puberty, in contrast with the significant impairment observed previously in PCOSr.

Other groups studying insulin dynamics in daughters or sisters of women diagnosed with PCOS during late pubertal stages of development relied primarily on the OGTT. A cross-sectional study [36] found that girls predisposed to PCOS (*n* = 92) displayed higher insulin levels 2 h–post OGTT, at any Tanner stage, than controls matched for age and BMI (*n* = 76). Similar results were found in another study using salivary insulin levels (*n* = 17 and 21, respectively) [37]. Sir-Petermann et al. [11, 12] also observed higher 2 h–insulin levels at any Tanner stage in PCOS first-degree relatives as compared to controls of the same BMI. On the other hand, Torchen et al. [14] observed similar insulin levels 2 h post-OGTT between PCOS first-degree relatives (*n* = 12) and controls (*n* = 10). In our study, 2-h insulin levels tended to be higher in PCOSr compared to control girls, but the ISI_Matsuda_ was similar between groups. However, insulin levels during OGTT and ISI_Matsuda_ are only estimations of insulin sensitivity, being less reliable than indices derived from the FSivGTT when compared to the gold standard hyperinsulinemic-euglycemic clamp in youth [28]. Furthermore, insulin clearance is reduced in PCOS women, compared to controls, which tends to increase insulinemia for the same degree of insulin sensitivity [38].

Consequently, we and others [14] have used FSivGTT to estimate systemic insulin sensitivity and *beta*-cell function in girls at risk of PCOS vs control girls. Torchen et al. [14] observed that *beta*-cell function remained lower over 2 years in PCOS at risk girls, from early to late pubertal stage (median Tanner stage of V at follow-up), compared to controls matched for age and BMI z-score. These results are similar to our baseline finding in this population [15]. On the other hand, insulin sensitivity was persistently similar between groups in Torchen’s study, as opposed to our baseline results. This may be explained by the fact that our PCOSr girls had higher adiposity than controls at baseline. This suggests that during early puberty, girls at risk for PCOS are characterized by *beta*-cell dysfunction independent of adiposity, as shown in two different populations. Insulin sensitivity is more likely related to girls’ adiposity.

We report the first cohort study on the evolution of glucose metabolism between early/mid puberty and late/post puberty in girls at risk for PCOS vs controls. Our study found significantly different changes in WHtR z-score between groups during this period (*p* = 0.05), explained by a decrease in PCOSr girls and an increase in controls. But even after correction for this change in WHtR z-score, we found that *beta*-cell function and insulin sensitivity deteriorated more in the control than the PCOSr groups. Consequently, glucose homeostasis of control girls matches PCOSr girls at the end of follow-up. Results of our control group may be explained by the normal transient insulin resistance observed during puberty. Using glucose-insulin camp techniques, Hannon and colleagues [16] observed a 50% decrease of insulin sensitivity in girls with normal weight and Tanner stage of IV-V, in comparison to their pre-pubertal insulin levels independently of changes in adiposity. A decrease in our control group was therefore expected. For our PCOSr girls, it appears that defective *beta*-cell function and insulin sensitivity were already maximal at baseline, and thus could not deteriorate further. Interestingly, our results are supported by a recent study using a sheep model predisposed to PCOS [39]. Female offspring of prenatally testosterone-treated sheep displayed higher insulin-to-glucose ratio, an index of insulin resistance, as compared to control sheep before puberty, but no differences were observed between groups at post-puberty and early adulthood.

Our group uniquely measured NEFA suppressibility in girls at risk for PCOS, an assessment of insulin-mediated suppression of adipocyte lipolysis. This is important because defective NEFA suppression could lead to spillover of NEFA in non-adipose tissue, causing lipotoxicity [18]. Lipotoxicity is now established as a key factor in the development of insulin resistance and type 2 diabetes [18]. We previously published that PCOSr girls in their early-mid pubertal years were characterized by an important reduction in their insulin-mediated NEFA suppressibility compared to controls [15]. Now, we show that NEFA suppressibility did not significantly vary between early and late pubertal years in our PCOSr girls, in contrast to control girls, whose NEFA suppressibility indices decreased significantly. These results suggest that, similarly to glucose metabolism, the sensitivity of adipocytes to insulin-suppression of lipolysis decreases only in control girls during puberty, such that controls become similar to PCOSr girls by late or post-puberty. In addition to the impact of puberty, it could be argued that these results might be explained by the increase of central adiposity in control girls. However, no correlation between changes in WHtR z-score and NEFA indices was observed in our study.

The main pitfall of our study is the lack of power to detect small differences between groups, due to its small sample size. This concern is mitigated by the use of a robust and accurate method (FSivGTT). Significant results were obtained using robust statistical methods and are thus reliable. Although we assessed multiple parameters using two methods (OGTT and FSivGTT), our results are concordant within and between methods, which increases the validity of our results. Another limitation of our study is that 38% of girls in both groups were on hormonal contraception. Accordingly, we cannot report reliable results on sex hormone levels, clinical hyperandrogenism and oligo-amenorrhea. Diagnosis of PCOS in at-risk girls was therefore impossible. Furthermore, only 40% of our PCOS-related girls are expected to be diagnosed with PCOS at adulthood [6]. Hence, indicators of PCOS predisposition in our cohort could be diluted by the results of girls who will not develop PCOS. Nevertheless, important and significant metabolic alterations were found between PCOS at risk and control girls during early puberty by two different research team [14, 15], suggesting that our cohort of PCOS-related girls remains an appropriate model to assess early predisposing factors to PCOS.

## Conclusions

We found that early defects in both glucose and NEFA metabolism in girls genetically predisposed to PCOS remain stable throughout puberty, as compared with control girls whose metabolic parameters deteriorate significantly, such that both groups become similar by pubertal conclusion. Accordingly, the early pubertal period may represent a transient window of metabolic perturbations for girls predisposed to PCOS. This new knowledge suggests that efforts should be made to look for metabolic markers of PCOS development in girls at risk during the early pubertal phase, as opposed to late- or post-puberty. Since pubertal-associated insulin resistance resolves following puberty and PCOS adult women are more insulin resistant than control women, it is expected that glucose and NEFA metabolism in at-risk and controls will segregate again beyond young adulthood. A longer follow-up will be required to assess this evolution in girls predisposed to PCOS during adulthood. An ongoing multicenter prospective study with a larger cohort of girls having a first-degree relative diagnosed with PCOS is led by Dr. David H. Geller (Los Angeles).

## Abbreviations

AIRg: Acute insulin response to glucose
AUC: Area under the curve
BMI: Body mass index
CHUS: Centre hospitalier universitaire de sherbrooke
CIR30: Corrected insulin response to glucose at 30 min
DI: Disposition index
FSivGTT: Frequently sampled intravenous glucose tolerance test
IS: Insulin sensitivity
ISI_Matsuda_: Matsuda insulin sensitivity index
Ln(NEFA) Slope: Slope of the log-linear decrease of non-esterified fatty acids levels
NEFA: Non-esterified fatty acids
OGTT: Oral glucose tolerance test
PCOS: Polycystic ovary syndrome
PCOSr: Daughters and sisters of women with polycystic ovary syndrome
T50_NEFA_: Time required to suppress 50% of non-esterified fatty acids at time zero
WC: Waist circumference
WHtR: Waist-to-height ratio
